# Plasma D-dimer can predict malignant brain edema after thrombectomy in noncardioembolic acute cerebral infarction

**DOI:** 10.1186/s12883-026-05139-2

**Published:** 2026-07-03

**Authors:** Wensheng Zhang, Zhenqin Jiang, Yangchun Wen, Xiaojing Zhong, Minzhen Zhu, Li Ling, Weifang Xing

**Affiliations:** 1 Department of Neurology, Heyuan People’s Hospital, Guangdong Provincial People’s Hospital Heyuan Hospital, Heyuan, Guangdong Province China; 2 Department of Neurology, Shenzhen Hospital, Southern Medical University, Shenzhen, Guangdong Province China; 3 The Third School of Clinical Medicine, Southern Medical University, Shenzhen, Guangdong Province China; 4 Heyuan Key Laboratory of Molecular Diagnosis & Disease Prevention and Treatment, Doctors Station of Guangdong province, Heyuan People’s Hospital, Heyuan, Guangdong Province China; 5 Department of Neurology, Guangdong Heyuan Traditional Chinese Medicine Hospital, Heyuan, Guangdong Province China

**Keywords:** Plasma D-dimer, Noncardioembolic acute cerebral infarction, Mechanical thrombectomy, Malignant brain edema

## Abstract

**Objective:**

There is relatively little data on malignant brain edema (MBE) after mechanical thrombectomy (MT) in non cardioembolic acute cerebral infarction caused by anterior circulation large vessel occlusion. Our study attempts to investigate the incidence rate of MBE after successful MT of anterior circulation acute non cardioembolic cerebral infarction, the predictive effect of emergency preoperative plasma D-dimer and clinical prognosis of patients.

**Methods:**

One hundred eighty-six consecutive patients suffered from anterior circulation non cardioembolic acute cerebral infarction who received successful MT were selected. Clinical features, imaging examinations, laboratory tests and treatment characteristics of patients at admission were collected and analyzed.

**Results:**

Among 186 patients (aged 64.26 ± 11.62 years; male accounted for 75.81%), 30 cases (16.13%) developed MBE. MBE patients had a low 3-month good prognosis rate and a high mortality rate (*P*<0.001). After adjusting for confounding factors, ASPECT score at admission (OR=0.322; 95% CI 0.146-0.711; *P*=0.005), tandem lesions in the location of occluded vessel (OR=6.422; 95% CI 1.799-22.924; *P*=0.004), collateral circulation score (OR=0.343; 95% CI 0.139-0.845; *P*=0.020) and emergency preoperative plasma D-dimer (OR=1.155; 95% CI 1.004-1.329; *P*=0.044) were significantly related to MBE. The area under the ROC curve of predicting MBE using emergency preoperative plasma D-dimer was 0.706 (sensitivity 0.500; specificity 0.859).

**Conclusion:**

Emergency preoperative plasma D-dimer is associated with MBE in patients with noncardioembolic acute cerebral infarction of anterior circulation after successful MT, although its predictive performance is modest. The presence of MBE reduces the chance of patients' neurological independence at 3 months after MT. These findings are preliminary and require validation in larger prospective studies.

## Introduction

Mechanical thrombectomy was proven to be the one of the most important treatment methods of acute cerebral infarction due to large vessel occlusion by many previous clinical studies [1]. However, even though that the occluded blood vessel was successfully recanalized, there was about still 50% of patients that cannot achieve nerve function independence 3 months after mechanical thrombectomy (MT), indicating that the patient’s occluded blood vessel recanalization was futile [2–4]. Complications such as malignant cerebral edema (MBE) after successful mechanical thrombectomy can weakening the therapeutic effect of MT [5–6]. If a patient suffers from MBE after receiving MT treatment, it can cause increased intracranial pressure, brain herniation and rapid deterioration of neurological function. Therefore, MBE is a relatively severe complication.

It had been found that age, NIHSS score at onset, preoperative ASPECT score, blood glucose level, collateral circulation score, and reperfusion grade were independent risk factors for MBE in patients with acute large vessel occlusion cerebral infarction after successful MT by previous studies [7–10]. However, to our knowledge, there is a relative lack of data on the incidence, effective predictive factors, and clinical prognosis of MBE after successful MT in acute cerebral infarction with noncardioembolic large vessel occlusion currently. It is still unclear whether there are independent emergency preoperative laboratory testing indicators that can influence the occurrence of MBE after successful mechanical thrombectomy. Plasma D-dimer is a product of fibrinogen degradation, whose content is within a certain range in healthy individuals. However, when thrombosis, high fibrinolysis, and infection happen, its content will routinely increase significantly [11–13]. We believe that plasma D-dimer may be an important factor of predicting MBE after successful MT for anterior circulation noncardioembolic acute cerebral infarction.

In addition, recent researches studying acute non cardiogenic cerebral infarction mostly concentrate on non‑large‑vessel occlusion [14–17]. Given the above reasons, this study preliminarily explores the incidence of MBE after successful MT of anterior circulation non cardiogenic large vessel occlusion in acute cerebral infarction, and the predictive effect of emergency preoperative plasma D-dimer in MBE.

## Materials and methods

### Study population

Data of patients who received successful MT for anterior circulation non cardiogenic acute cerebral infarction from January 2019 to December 2023 was collected in Heyuan People’s Hospital. The study plan of this research had been approved by the Medical Ethics Committee of Heyuan People’s Hospital. Taking into account that our study only collected data and conducted retrospective analysis without intervening in patients’ treatment plans, the medical ethics committee agree to waive written informed consent for this study.

### Inclusion and exclusion criteria

**Fig. 1:**
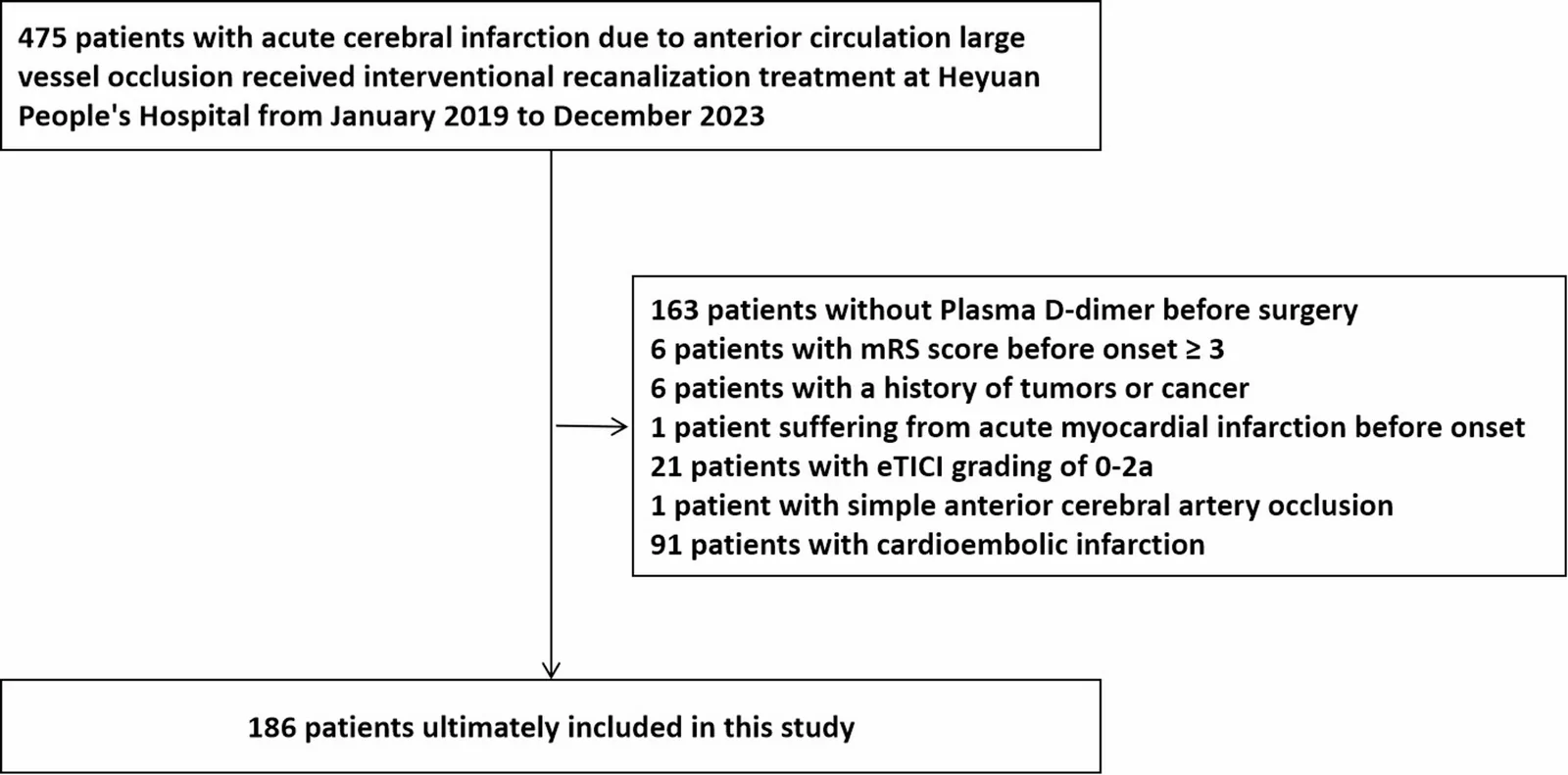
Research flowchart. eTICI: extended thrombolysis in cerebral infarction

Inclusion of this study were as follows: (1) Patient age was ≥ 18 years old; (2) Prior to MT, imaging data confirmed that the patient suffered from anterior circulation large vessel occlusion, including the internal carotid artery, middle cerebral artery M1 segment, and middle cerebral artery M2 segment; (3) At admission, the patient’s National Institutes of Health Stroke Scale (NIHSS) score was ≥ 6 points [18]; (4) The Alberta Stroke Project Early Computed Tomography (ASPECT) score of the patient’s preoperative brain CT was ≥ 6 points [19]; (5) The pre-onset mRS score of the patient was ≤ 2 points [20]; (6) Time from onset to femoral artery puncture was ≤ 24 h; (7) The patient or their family members agreed to undergo mechanical thrombectomy; (8) According to patients’ preoperative clinical symptoms, onset characteristics, imaging examination features, intraoperative cerebral angiography results, postoperative dynamic electrocardiogram, and other further examinations, it was confirmed that the patient’s pathogenesis was non cardioembolic embolism. Exclusion criteria of this study were as follows: (1) The patient was confirmed to be suffered from posterior circulation large vessel occlusion; (2) The patient had received intravenous thrombolysis treatment prior to collecting blood samples; (3) The patient’s modified Rankin Scale (mRS) score before onset was greater than 2 points; (4) The patient had diseases that may influence plasma D-dimer level, such as acute myocardial infarction, deep vein thrombosis, pulmonary embolism and active cancer; (5) The patient’s blood sample was not collected before mechanical thrombectomy; (6) The patient’s pathogenesis was considered to be cardiac embolism; (7) The patient’s vital signs were unstable at onset. The flowchart of this study is shown in Fig. 1.

### Disposion and detection of plasma D-dimer

Patients who were considered to have acute cerebral infarction will routinely to do coagulation function test at emergency department. After collecting the peripheral venous blood of each patient, blood sample will be placed into a blood collection tube containing sodium citrate as an anticoagulant. The centrifugation time for venous blood samples is 10 min, and the centrifugation speed is 3000 revolutions per minute. The fully automatic biochemical detector (Stago Emo Express instrument, manufacturer: Stago, France) was used to detect plasma D-dimer in patients, and the detection method was immunoturbidimetry.

### MT methods

The methods of mechanical thrombectomy in this study included stent thrombectomy, thrombus aspiration, balloon dilation, stent implantation, and arterial injection of tirofiban injection. Interventional physicians used one or more of the above methods to mechanically remove thrombi during surgery according to the pathogenesis and path of the patient’s lesion.

### Details of data collection

We collected data including demographic information of patients, laboratory test indicators, NIHSS score, preoperative ASPECT score, collateral circulation score, and characteristics of surgical procedure. The assessment of collateral circulation was carried out using the American Society of Interventional Neuroradiology/Society of Interventional Radiology (ASTIN/SIR) scoring system [21]. The characteristic of the surgical process included whether tirofiban was injected into the artery, the time from onset to arrival at the hospital, and the time from femoral artery puncture to recanalization of occluded blood vessel. The assessment of recanalization of occluded blood vessel was carried out using the extended Thrombolysis for Cerebral Infarction (eTICI) blood flow grading system [22].

### Assessment of MBE

Non-enhanced CT was routinely performed immediately after MT and at 24 h post-MT. Additional non-enhanced CT or MRI was obtained between 24 and 72 h after MT based on the patient’s clinical condition; immediate repeat imaging was performed if neurological deterioration occurred.

The definition of MBE was pre-specified based on published criteria [8] and required both of the following objective measures: (1) hypodensity involving ≥ 50% of the middle cerebral artery (MCA) territory with evidence of local brain swelling (e.g., sulcal effacement or compression of the lateral ventricle); and (2) midline shift (of the septum pellucidum or pineal gland) of ≥ 5 mm measured on axial CT images, accompanied by effacement of the basal cisterns. All measurements were performed using digital calipers on the institutional picture archiving and communication system system.

MBE was evaluated independently by two neuroradiologists who were blinded to all clinical and laboratory data. In the event of disagreement (which occurred in 4 cases, 2.2%), a third senior neuroradiologist reviewed the images and the final diagnosis was reached by majority consensus.

### Follow-up after mechanical thrombectomy

At 3 months after the patient underwent MT, we followed up with the patient by phone and scored their mRS score. Patients with an mRS score of less than or equal to 1 had an excellent prognosis, patients with an mRS score of 0 to 2 had a good prognosis, patients with an mRS score greater than 2 had a poor prognosis, and patients with an mRS score of 6 had already died.

### Efforts to mitigate limitations of the retrospective design

To minimize the inherent biases of a retrospective study, the following measures were implemented: (1) Consecutive enrollment of all eligible patients during the study period to reduce selection bias; (2) Use of a pre-defined standardized case report form for data extraction; (3) Blinding of outcome assessors (neuroradiologists for MBE assessment and follow-up interviewers for mRS scores) to plasma D-dimer levels and other laboratory results; (4) Multivariate logistic regression analysis to adjust for potential confounders; (5) Pre-specified inclusion and exclusion criteria to ensure homogeneity of the study population. These strategies were designed to improve the internal validity of the findings despite the retrospective nature of the study.

### Statistical analysis

We used mean ± standard deviation or median (interquartile range) to represent measurement data. We perform statistical analysis on measurement data that conformed to normal distribution using t-test, and perform statistical analysis on measurement data that did not conform to normal distribution using rank sum test. We used frequency and percentage to represent count data. Chi square test was used for statistical analysis on count data. In univariate analysis, factors associated with MBE (*P* < 0.05) were entered into a multivariate logistic regression model using a forward stepwise (conditional) selection method to identify independent risk factors for MBE. Based on the results of multiple regression analysis, we drew a forest plot of the relative risk of MBE. In addition, the cutoff value, sensitivity, and specificity of predictive indicators were calculated by the receiver operating characteristic (ROC) curve. The statistical software we used was SPSS 26.0.

## Results

### Baseline clinical characteristic

The average age of all patients was 64.26 ± 11.62 years, with 141 males (75.81%). At the onset of the disease, the NIHSS score was 11.00 (8.75-15.00), ASPECT score at admission was 8.00 (7.00–9.00), the collateral circulation score was 3.00 (2.00–3.00) and the time from onset to recanalization of occluded blood vessels was 577.00 (434.75–823.50) minutes. The baseline clinical data of patients with MBE and those without MBE are shown in Table 1. All patients underwent a 3-month follow-up after MT. 105 patients (56.45%) achieved independent neurological function 3 months after MT and 8 patients (4.3%) died within 3 months after MT. The prognosis of patients with MBE and those without malignant brain edema at 3 months after MT is shown in Table 2.

**Table 1:** Univariate analysis of MBE in patients with acute cerebral infarction and received successful MT due to non cardio embolism large vessel occlusion in the anterior circulation

Variables	MBE (*n* = 30)	No MBE (*n* = 156)	*P* value
Age, years, (mean ± standard deviation)	62.77 ± 13.30	64.55 ± 11.29	0.413
Gender, male, n (%)	20(66.67)	121(77.56)	0.202
History of medicine			
Hypertension, n (%)	14(46.67)	88(56.41)	0.326
Diabetes, n (%)	8(26.67)	32(20.51)	0.452
Cerebral infarction, n (%)	5(16.67)	21(13.46)	0.643
Coronary heart disease, n (%)	1(3.33)	14(8.97)	0.299
Atrial fibrillation, n (%)	0(0)	2(1.28)	0.533
Smoking history, n (%)	13(43.33)	70(44.87)	0.877
Drinking history, n (%)	7(23.33)	40(25.64)	0.790
Pre onset mRS score, (mean ± standard deviation)	0.13 ± 0.57	0.08 ± 0.34	0.934
The situation at the onset of stroke			
NIHSS score at admission, median (IQR)	15.50(10.75-18.00)	11.00(8.00–14.00)	**<0.001**
ASPECT score at admission, median (IQR)	6.00(6.00–7.00)	8.00(7.00–9.00)	**<0.001**
Received intravenous thrombolysis treatment, n (%)	13(43.33)	51(32.69)	0.261
Location of occluded blood vessels, n (%)			**0.002**
M1 segment of middle cerebral artery	7(23.33)	76(48.72)	
M2 segment of middle cerebral artery	1(3.33)	12(7.69)	
Internal carotid	3(10.00)	26(16.67)	
Anterior circulation tandem lesion	19(63.33)	42(26.92)	
Collateral circulation score, median (IQR)	2.00(1.00–2.00)	3.00(2.00–3.00)	**<0.001**
Intraarterial use of tirofiban injection, n (%)	6(20.00)	57(36.54)	0.080
eTICI grading of reperfusion blood flow, n (%)			**0.021**
2b	10(33.33)	23(14.74)	
2c	3(10.00)	8(51.28)	
3	17(56.67)	125(80.13)	
Time node			
Time from arrival to femoral artery puncture, min, median (IQR)	132.50(95.00-151.00)	122.00(101.50–163.00)	0.565
Time from puncture to recanalization of the occluded vessel, min, median (IQR)	114.00(82.50-158.25)	100.00(77.25-129.75)	0.117
Time from onset to recanalization of the occluded blood vessel, min, median (IQR)	600.00(453.75-966.75)	571.50(423.00-786.25)	0.523
Laboratory test results			
Platelet count, 10 ^ 9/L, median (IQR)	206.50(164.75–241.00)	221.50(186.50–264.00)	0.130
Plasma D-dimer, ug/ml, median (IQR)	1.715(0.670–5.645)	0.705(0.368–1.348)	**<0.001**
Fibrinogen, g/L, median (IQR)	3.105(3.253–3.728)	3.220(2.723–3.680)	0.380

*MBE* Malignant brain edema, *MT* mechanical thrombectomy, *NIHSS* National Institute of Health stroke scale, *ASPECT* Alberta Stroke Program Early Computed Tomography, *eTICI* extended thrombolysis in cerebral infarction

**Table 2:** Bone flap decompression surgery and prognostic differences between patients with MBE and those without MBE after MT for acute cerebral infarction with non cardio embolism large vessel occlusion of anterior circulation

Variables	MBE (*n* = 30)	No MBE (*n* = 156)	*P* value
Bone flap decompression surgery, n (%)	3(10.00)	0(0)	**<0.001**
mRS score 3 months after MT, median (IQR)	4.00(3.00–6.00)	2.00(1.00–4.00)	**<0.001**
mRS score 3 months after MT ≤ 2 points, n (%)	2(6.67)	103(66.03)	**<0.001**
mRS score 3 months after MT ≤ 1 points, n (%)	1(3.33)	75(48.08)	**<0.001**
Death within 3 months after MT, n (%)	8(26.67)	0(0)	**<0.001**

*MBE* Malignant brain edema, *MT* mechanical thrombectomy, *mRS* modified Rankin scale

### The incidence and related factors of MBE

Among all patients, 30 patients (16.13%) developed MBE. It was found that compared with patients without MBE, patients with MBE had higher NIHSS scores at onset (15.50 vs. 11.00; *P* < 0.001), lower ASPECT score at admission (6.00 vs. 8.00; *P* < 0.001), higher proportion of anterior circulation tandem lesions in location of occluded vessels (63.33% vs. 26.92%; *P* = 0.002), lower collateral circulation scores (2.00 vs. 3.00; *P* < 0.001), lower proportion of reperfusion blood flow grades 2c and 3 (2c: 10.00% vs. 51.28%; 3:56.67% vs. 80.13%; *P* = 0.021) and higher plasma D-dimer levels (1.715ug/ml vs. 0.705ug/ml; *P* < 0.001) in univariate analysis. Univariate analysis is shown in Table 1.

**Fig. 2:**
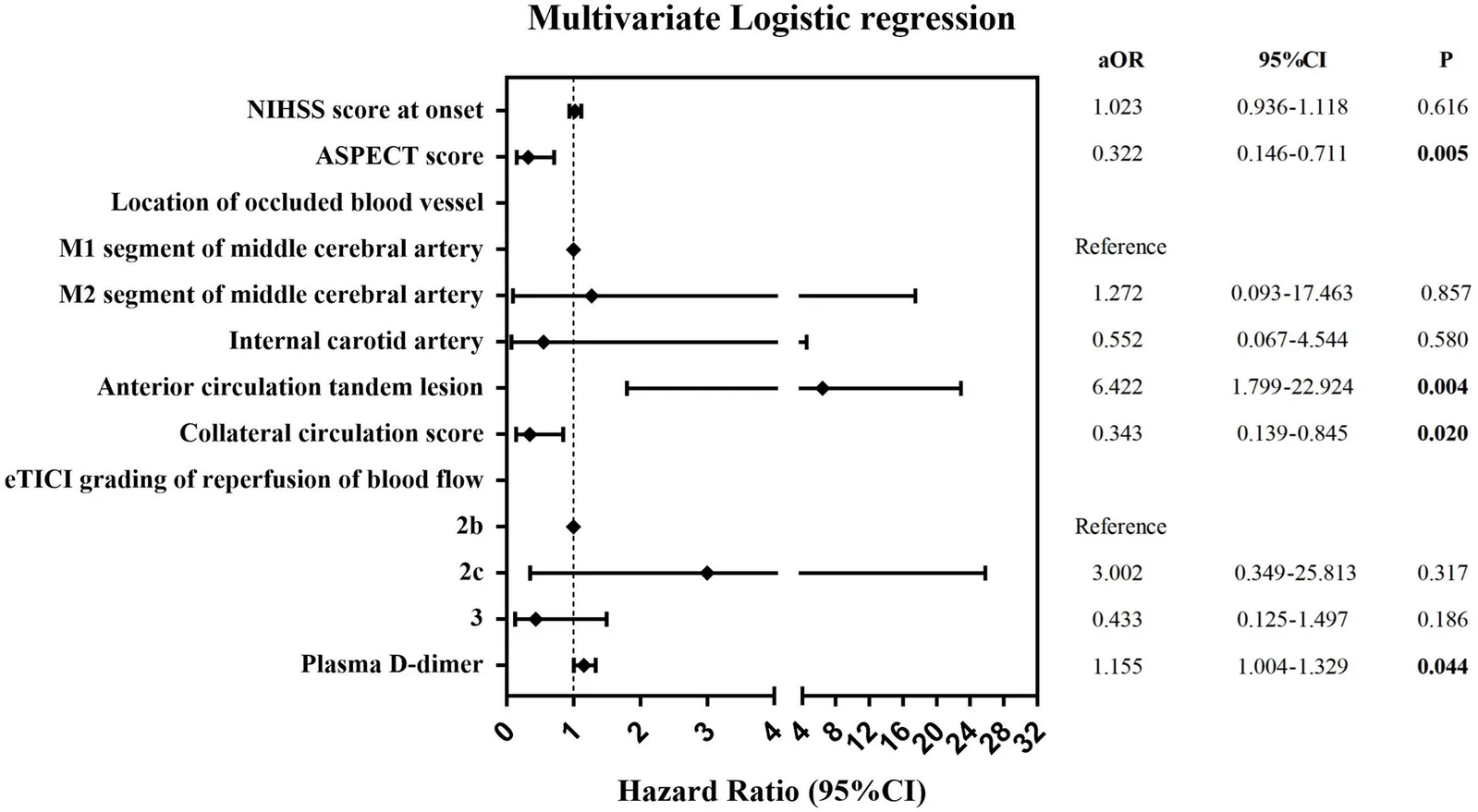
Forest plot of the relative hazard ratios of MBE corresponding to each indicator in multiple logistic regression analysis. NIHSS: National Institute of Health stroke scale; ASPECT: Alberta Stroke Program Early Computed Tomography; eTICI: extended thrombolysis in cerebral infarction

After adjusting for confounding factors, it was found that ASPECT score at admission (OR = 0.322; 95% CI 0.146–0.711; *P* = 0.005), anterior circulation tandem lesions in the location of occluded vessel (OR = 6.422; 95% CI 1.799–22.924; *P* = 0.004), collateral circulation score (OR = 0.343; 95% CI 0.139–0.845; *P* = 0.020) and plasma D-dimer levels (OR = 1.155; 95% CI 1.004–1.329; *P* = 0.044) were independently associated with MBE in multivariate logistic regression analysis. Multivariate logistic regression analysis is shown in Table 3. The forest plot of the hazard ratios of malignant brain edema corresponding to each indicator analyzed in multiple logistic regression is shown in Fig. 2.

**Table 3:** Univariate and multivariate logistic regression analysis of MBE patients after successful MT for acute cerebral infarction with anterior circulation non cardio embolism

Variables	Univariate analysis				Multivariate analysis		
	OR	95%CI	*P* value		OR	95%CI	Pvalue
NIHSS score at admission	1.126	1.052–1.206	**0.001**		1.023	0.936–1.118	0.616
ASPECT score at admission	0.205	0.112–0.376	**<0.001**		0.322	0.146–0.711	**0.005**
Location of occluded blood vessels							
M1 segment of middle cerebral artery	Reference				Reference		
M2 segment of middle cerebral artery	0.905	0.102–8.019	0.928		1.272	0.093–17.463	0.857
Internal carotid	1.253	0.302–5.203	0.756		0.552	0.067–4.544	0.580
Anterior circulation tandem lesion	4.912	1.909–12.636	**0.001**		6.422	1.799–22.924	**0.004**
Collateral circulation score	0.172	0.091–0.325	**<0.001**		0.343	0.139–0.845	**0.020**
eTICI grading of reperfusion blood flow							
2b	Reference				Reference		
2c	0.863	0.189–3.945	0.849		3.002	0.349–25.813	0.317
3	0.313	0.127–0.768	**0.011**		0.433	0.125–1.497	0.186
Plasma D-dimer	1.142	1.043–1.251	**0.004**		1.155	1.004–1.329	**0.044**

*MBE* Malignant brain edema, *MT* mechanical thrombectomy, *NIHSS* National Institute of Health stroke scale, *ASPECT* Alberta Stroke Program Early Computed Tomography, *eTICI* extended thrombolysis in cerebral infarction

**Fig. 3:**
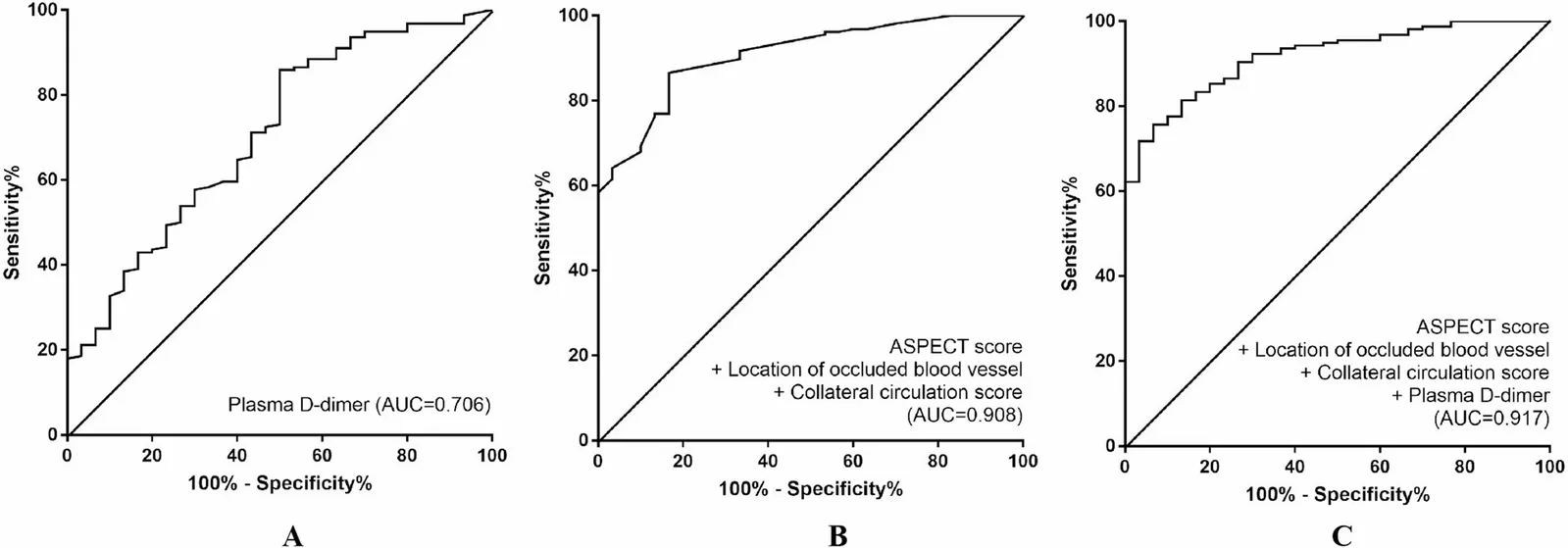
**A** ROC curve for predicting malignant brain edema using emergency preoperative plasma D-dimer level. **B** ROC curve for predicting MBE using a combination of three indicators including ASPECT score at admission, location of occluded blood vessels and collateral circulation score. **C** ROC curve for predicting MBE using a combination of four indicators including emergency preoperative plasma D-dimer, ASPECT score at admission, location of occluded blood vessels and collateral circulation score.ROC: Receiver Operating Characteristic; ASPECT: Alberta Stroke Program Early Computed Tomography; AUC: area under the curve

The ROC curve for predicting MBE based on plasma D-dimer levels was drawn with an area under the curve (AUC) of 0.706 (cut-off value: 2.065ug/ml, sensitivity: 50.00%; specificity: 85.90%). The ROC curve for predicting malignant brain edema using three combined indicators including ASPECT score at admission, occluded vessel location and collateral circulation score was drawn with an AUC of 0.908 (sensitivity: 83.30%; specificity: 86.50%). The ROC curve for predicting malignant brain edema using four combined indicators including preoperative ASPECT score, occluded vessel location, collateral circulation score and plasma D-dimer level was drawn with an AUC of 0.917 (sensitivity: 93.30%; specificity: 75.60%). The ROC curves are shown in Figs. 3A, 3B, and 3C.

When it came to decompressive hemicraniectomy, 3 cases (10.00%) of patients in the MBE group underwent bone flap decompression surgery, while no patients in the non MBE group underwent decompressive hemicraniectomy. There was a statistically significant difference between the two groups (*P* < 0.001). The statistical process is shown in Table 2.

### The correlation between MBE and prognosis

**Fig. 4:**
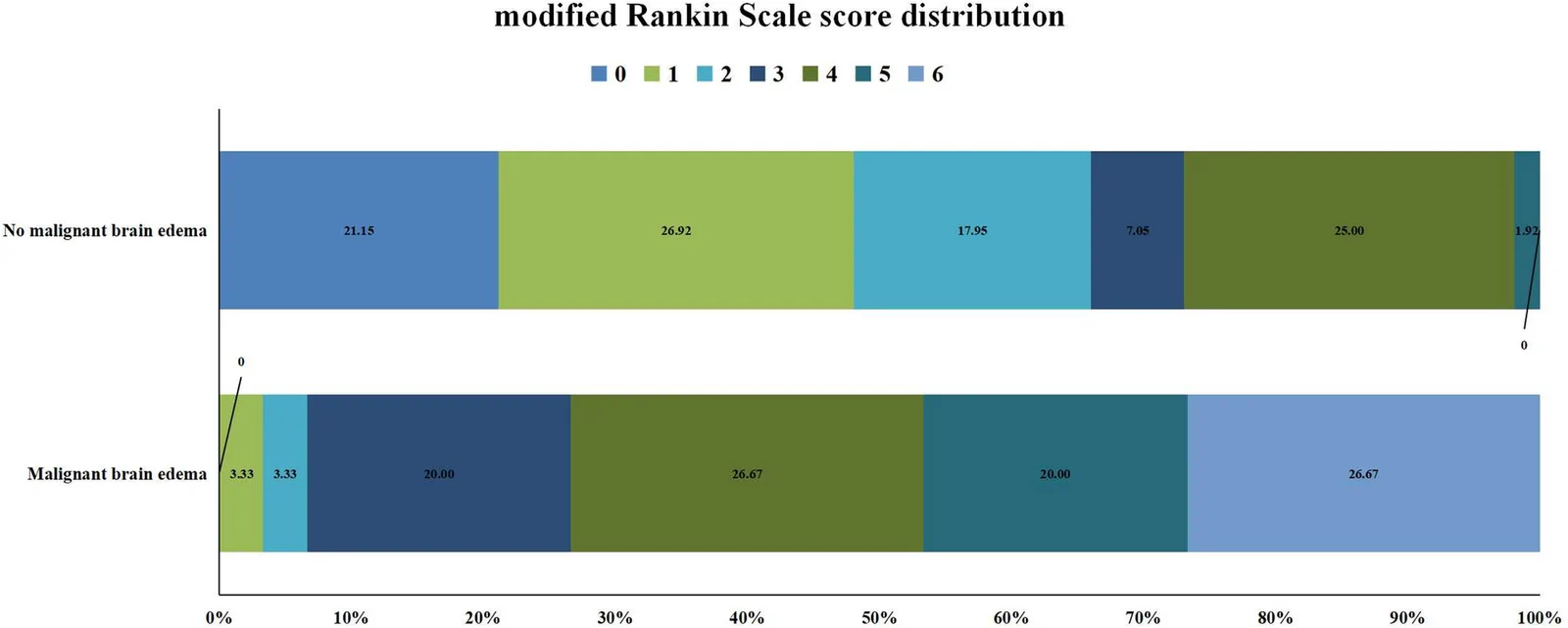
Distribution of mRS scores at 3 months after MT of two groups of patients with MBE and those without MBE.mRS: modified Rankin scale

In univariate analysis, compared with patients without MBE, patients with MBE had a lower proportion of independent neurological function (mRS score ≤ 2) at 3 months after surgery (6.67% vs. 66.03%; *P* < 0.001), a higher mRS score at 3 months after surgery (4.00 vs. 2.00; *P* < 0.001), a lower proportion of excellent prognosis (mRS score ≤ 1) at 3 months after MT (3.33% vs. 48.08%; *P* < 0.001) and a higher mortality rate at 3 months after MT (26.67% vs. 0; *P* < 0.001). The distribution of mRS scores at 3 months after MT in patients with MBE and those without MBE is shown in Fig. 4.

## Discussion

In our study, we explored the incidence, risk factors and clinical outcomes of MBE in patients suffered from anterior circulation noncardioembolic acute cerebral infarction after successful MT. The main findings are as follows. Firstly, MBE is not uncommon after successful MT in noncardioembolic acute cerebral infarction. Secondly, plasma D-dimer level, ASPECT score at admission, location of occluded vessels and collateral circulation scores are independent predictive factors for MBE. Thirdly, the presence of MBE can reduce the chance of patients’ neurological independence and increase the mortality rate at 3 months after MT.

Currently, researches on noncardioembolic acute cerebral infarction mainly concentrated on mild stroke without large vessel occlusion, and there are few studies aim to explore the effectiveness and safety of MT in non cardioembolic acute anterior circulation large vessel occlusion cerebral infarction [14–17]. Noncardioembolic acute large vessel occlusion cerebral infarction, as an important type of acute cerebral infarction, accounts for more than 50% of all acute cerebral infarction due to large vessel occlusion of anterior circulation. Noncardioembolic pathogenesis mainly includes large artery atherosclerosis and vascular dissection [23]. Unlike acute cerebral infarction caused by cardiac embolism, the characteristic of a large proportion of patients with acute noncardioembolic large vessel occlusion cerebral infarction may be worsening gradually [24]. We believe that it is of importantance to explore the treatment of acute cerebral infarction with non cardio embolism large vessel occlusion separately.

Multicenter clinical studies had previously confirmed that MT was effective and safe for acute cerebral infarction caused by occlusion of large blood vessels of anterior circulation. After successful MT, nearly 50% of patients still cannot achieve neurological function independence within 3 months after MT, which was called futile recanalization [25]. Symptomatic cerebral hemorrhage and MBE are serious complications after MT, and they affect the effectiveness of MT of patients [7, 26]. MBE is mainly characterized by transparent septum or pineal midline displacement of ≥ 5 mm within 72 h after mechanical thrombectomy, basal cistern occlusion, accompanied by cerebral parenchymal low-density in MCA areas of ≥ 50%, and local cerebral swelling, such as thinning of cerebral sulci and compression of lateral ventricles [8]. It had been founded that the incidence of MBE after MT in acute anterior circulation large vessel occlusion cerebral infarction is approximately 10% -27% [8]. Our study found that the incidence of MBE after successful MT in noncardioembolic acute cerebral infarction with anterior circulation large vessel occlusion was 16.13%. Our study had some differences in the incidence of MBE compared to previous studies, which may be related to the reason that we only studied the pathogenesis of noncardioembolic stroke.

It had been confirmed that MBE was associated with futile recanalization and increased mortality rate 3 months after MT [8, 27]. Our study also found that in patients with successful MT of noncardioembolic acute cerebral infarction of anterior circulation, MBE is associated with futile recanalization and increased mortality rate at 3 months after MT. At the same time, our study also found that the proportion of patients with MBE who had excellent prognosis at 3 months after MT was significantly lower than that of patients without MBE. Currently, there are many studies on symptomatic cerebral hemorrhage after MT, and the exploration is also relatively in-depth [28–30]. However, there are few studies exploring the use of preoperative coagulation function indicators or other laboratory test indicators to predict MBE. It was founded previously that age, NIHSS score at admission, ASPECT score at admission, blood glucose level, collateral circulation score, and reperfusion grading were independent risk factors of MBE after MT [7–10]. A study had also found that emergency preoperative plasma D-dimer level can predict symptomatic cerebral hemorrhage after MT [26]. However, there is no literature reporting that emergency preoperative plasma D-dimer can predict MBE after successful MT in acute cerebral infarction.

The content of plasma D-dimer is within a certain range in healthy individuals. However, plasma D-dimer would significantly increase in the situation of thrombosis, high fibrinolysis and infection [11–12]. It had been proved that the elevated plasma D-dimer level was related to pulmonary embolism, deep vein thrombosis, active cancer and acute myocardial infarction [27–29]. Our research ultimately found that the level of emergency preoperative plasma D-dimer can effectively predict MBE after successful MT in noncardioembolic acute cerebral infarction of anterior circulation. This could help to screen patients who have a better chance of benefiting from MT in the very early stage, and also could help to develop postoperative management plans in the very early stage, such as early use of dehydration drugs, early craniotomy and decompressive hemicraniectomy, and selection of high head position after MT, so as to reduce intracranial pressure and minimizing the harm of MBE.

D-dimer is a fibrin degradation product whose elevation reflects activation of both coagulation and fibrinolysis [11]. In acute ischemic stroke, elevated D-dimer had been reported to be associated with blood-brain barrier (BBB) disruption via matrix metalloproteinase (MMP) activation [7, 10] Specifically, MMP-9 degrades tight junction proteins of the BBB [31], leading to increased vascular permeability—a key step in the pathogenesis of vasogenic edema [10]. Furthermore, elevated D-dimer levels are often accompanied by an inflammatory response involving cytokines such as interleukin-6, which further compromise BBB integrity and promote edema formation [6, 11, 14]. In patients undergoing successful mechanical thrombectomy, reperfusion itself may paradoxically exacerbate these inflammatory and edematous processes [5, 13]. Therefore, higher baseline D-dimer levels may identify patients with more severe endothelial injury and a greater propensity for MBE after successful recanalization.

In addition to plasma D-dimer, our study also found that independent risk factors of MBE included low ASPECT score at admission, low collateral circulation score and the location of occluded blood vessels being anterior circulation tandem lesions. A lower ASPECT score may present a larger volume and a greater number of infarcted sites. A lower collateral circulation score may present a faster volume growth rate in cerebral infarction. Tandem lesions means that more blood vessels are involved, and a larger volume of cerebral infarction, and the process of MT may be more difficult.

Our study had certain limitations. First, this was a single-center, retrospective study, which inherently carries a risk of selection bias and limits the generalizability of our findings. Second, a key limitation is the relatively small number of MBE events (*n* = 30), which raises concerns about statistical stability and potential overfitting in the multivariate model. Consequently, our findings should be interpreted as exploratory. Third, we did not perform internal (e.g., bootstrap resampling) or external validation of our predictive model, which further underscores the preliminary nature of our conclusions. Fourth, some patients did not undergo plasma D-dimer testing before MT, which may introduce selection bias. Fifth, despite adjusting for several confounders (age, baseline NIHSS, glucose level, ASPECT score, collateral circulation, and tandem lesions), we cannot rule out residual or unmeasured confounding factors, such as detailed inflammatory markers or the use of specific anti-edema medications. In the future, a larger sample size, multicenter, prospective study with internal and external validation is needed to further confirm our findings. However, to our knowledge, this study is the first to explore the predictive role of emergency preoperative plasma D-dimer in MBE.

## Conclusion

Emergency preoperative plasma D-dimer is associated with MBE in patients with noncardioembolic acute cerebral infarction of anterior circulation after successful MT, although its predictive performance is modest. The presence of MBE reduces the chance of patients’ neurological independence at 3 months after MT. These findings are preliminary and require validation in larger prospective studies. 

## Data Availability

The datasets analyzed during the current study are not publicly available due to intellectual property rights, but are available from the corresponding author on reasonable request.

## Abbreviations

MBE: Malignant brain edema
MT: Mechanical thrombectomy
NIHSS: National Institute of Health stroke scale
ASPECT: Alberta Stroke Program Early Computed Tomography
mRS: Modified Rankin scale
ASTIN/SIR: American Society of Interventional and Therapeutic Neuroradiology/ Society of Interventional Radiology
eTICI: Extended thrombolysis in cerebral infarction
ROC: Receiver Operating Characteristic
AUC: Area under the curve
